# Molecular Characterization of Apricot Germplasm from an Old Stone Collection

**DOI:** 10.1371/journal.pone.0023979

**Published:** 2011-08-25

**Authors:** Carolina Martín, María Herrero, José I. Hormaza

**Affiliations:** 1 IHSM “La Mayora” - CSIC, Algarrobo-Costa, Málaga, Spain; 2 Pomology Department, EE Aula Dei, CSIC, Zaragoza, Spain; University of Melbourne, Australia

## Abstract

Increasing germplasm erosion requires the recovery and conservation of traditional cultivars before they disappear. Here we present a particular case in Spain where a thorough prospection of local fruit tree species was performed in the 1950s with detailed data of the origin of each genotype but, unfortunately, the accessions are no longer conserved in *ex situ* germplasm collections. However, for most of those cultivars, an old stone collection is still preserved. In order to analyze the diversity present at the time when the prospection was made and to which extent variability has been eroded, we developed a protocol in apricot (*Prunus armeniaca* L.) to obtain DNA from maternal tissues of the stones of a sufficient quality to be amplified by PCR. The results obtained have been compared with the results from the profiles developed from apricot cultivars currently conserved in *ex situ* germplasm collections. The results highlight the fact that most of the old accessions are not conserved *ex situ* but provide a tool to prioritize the recovery of particular cultivars. The approach used in this work can also be applied to other plant species where seeds have been preserved.

## Introduction

The development of new technologies, the substitution of local varieties by foreign improved varieties or changes in cultural techniques have resulted in an increasing erosion of germplasm resources that leads to the need of optimizing the conservation of endangered germplasm [1]. In fact, conservation and use of plant genetic resources should be a priority in agricultural research [2]-[5]. However, this task is often hindered by the abundance of homonymies and synonymies in germplasm collections and the lack of information available on local germplasm erosion.

In this work we present a case study in apricot (*Prunus armeniaca* L.) in Spain. Apricot is an economically important member of the Rosaceae cultivated in Mediterranean climates worldwide. Apricot is a diploid species, with eight pair of chromosomes (2n = 16) and a small genome (5.9×10^8^ bp) [6] that is believed to have originated in the Tien-Shan Mountains, in Central Asia, from where it was disseminated both east and westward [7]. The species can be classified into six main ecogeographical groups [8]: Central Asian, East Chinese, North Chinese, Dzhungar-Zailij, Irano-Caucasian and European. However, due the introduction of new cultivars derived from crosses between genotypes of the different groups, the assignment of new cultivars to one of these groups is difficult [9]. In the last ten years a clear effort has been made to characterise apricot germplasm in different parts of the world [10]–[19] generally showing a regional distribution that probably reflects independent selection in each region and later vegetative propagation of selected genotypes through grafting.

Apricot was introduced in the Mediterranean region from Iran or Armenia around the first century BC [20], although more recently new introductions were made from the Middle East, especially into Southern Europe [9]. Spanish apricot cultivars could have been derived from genotypes of both the European and the Irano-Caucasian groups, the latter introduced from Northern Africa by the Arabs [21]. Under that scenario we would expect a high level of variability among the Spanish cultivars; however, this is not the case [10], [22] and probably reflects an erosion of the variability present in the Spanish cultivars due to the small geographical area where apricot has been traditionally cultivated in Spain, to the generalized use of grafting in the last two centuries and to the predominance of few cultivars such as ‘Búlida’, ‘Canino’ or ‘Moniquí’ that could be ancestors of most of the cultivars currently available in Spain [22].

One of the main limiting factors to analyze genetic erosion is the lack of knowledge on the genetic composition of the cultivars that have been lost, since for most of them we only have written records but it is not possible to ascertain if those cultivars have been preserved in *ex situ* collections under a different name. In this sense, a thorough inventory of cultivars of different fruit tree species, including apricot, was performed in Spain in the 1950s with detailed data of the collected site of each genotype [23]. Although most of the genotypes are no longer conserved, at least with the same name, in *ex situ* collections, old stones from some of those genotypes are still preserved. This situation is not particular of apricot but is generalized in a good number of woody perennials, since the stone collections were commonly used at that time for morphological identification purposes. Since the fruit of *Prunus* species is a drupe where the mature stony endocarp together with the seed forms a propagation unit, there are two tissues of maternal origin that should represent the genetic profile of these old cultivars: the endocarp that derives from the inner layer of the ovary, and the testa that derives from the integuments of the ovule. But we wondered if DNA extracted from this old material could still reveal a genetic profile and thus reflect the genetic variability present at the time of the collection in the field. Thus, as a first step to evaluate the loss of old apricot material in the last decades in Spain we optimized a method to extract DNA from two maternal tissues (the endocarp and the testa) of old stones of apricot to allow the fingerprinting of the old cultivars that originated these fruits. For molecular analyses we used Simple Sequence Repeat (SSR) markers that have been successfully used in apricot germplasm characterization in different works [10]–[19]. In a second step we evaluated the variability of this material in relation to cultivars currently preserved in *ex situ* collections. Results shed light on how to prioritize recovery of old cultivars.

## Materials and Methods

### Plant material

Two different local Spanish apricot sample sets were used in this work, one from an old apricot stone collection, and the other from young leaves of apricot cultivars conserved in *ex situ* living collections. Endocarps and seed testas collected from thirty four apricot genotypes in the 1950s from different geographical areas in Spain (Andalucia, Balearic Islands, Valencian Community, Murcia and Ebro Valley), and conserved at the E.E. Aula Dei in Zaragoza (Spain) at room temperature, and leaves from twenty four apricot accessions conserved *ex situ*, twenty one in the germplasm collection of CITA in Zaragoza (Spain) and three maintained in the E.E Aula Dei in Zaragoza (Spain), were analysed and compared in this study (**Table 1**).

**Table 1:** List of the Spanish apricot cultivars from the old stone and living *ex situ* collection.

STONE COLLECTION		*EX SITU* COLLECTION	
Cultivars	Prospection area	Cultivars	Origin
Acmé	Logroño; Ebro Valley	Berdejo	Zaragoza
Amoscatelado	Sabiñan, Zaragoza; Ebro Valley	Blancos	Valencia
Antón	Cieza; Murcia	Bulida AD	Murcia, Albacete
Blanco de Murcia 1	Logroño; Ebro Valley	Canino 1	Valencia
Blanco de Murcia 2	Sabiñan, Zaragoza; Ebro Valley	Canino 2	Valencia
Canino 1	Monzón, Huesca; Ebro Valley	Corbato 1	Valencia
Canino 2	Valencia; Valencian Community	Corbato 2	Valencia
Carmelos	Logroño; Ebro Valley	Cristali	Valencia
Damasco	Sabiñan, Zaragoza; Ebro Valley	Currot	Valencia
De Antón	Logroño; Ebro Valley	Ginesta	Valencia
De Confitar	Milagro, Logroño; Ebro Valley	Gitano AD	Valencia
De Hellín	Calatayud, Zaragoza; Ebro Valley	Merino	Unknown
Encarnado Fino	Sabiñan, Zaragoza; Ebro Valley	Mitjer 1	Valencia
Galta Vermeya	Porreras; Balearic Islands	Mitjer 2	Valencia
Giletano 1	Segorbe, Castellón; Valencian Community Community	Moniquí Borde AD	Murcia
Giletano 2	Carlet, Valencia; Valencian Community	Moniquí 1	Zaragoza, Albacete, Murcia
Gitano	Abarán; Murcia	Moniquí 2	Zaragoza, Albacete, Murcia
Hoja de Parra	Logroño; Ebro Valley	Muñoz	Unknown
Moniquí	Logroño; Ebro Valley	Peñaflor	Zaragoza
Moniquí Temprano	Jaén; Andalucia	Pepitos del Rubio 1	Murcia
Patriarca de Hueso Dulce	Segorbe, Castellón; Valencian Community	Pepitos del Rubio 2	Murcia
Perla	Logroño; Ebro Valley	Rojo de Carlet	Valencia
Precoz de Boulbon	Logroño; Ebro Valley	Tadeo 1	Valencia
Real Fino 1	Calatayud, Zaragoza; Ebro Valley	Tadeo 2	Valencia
Real Fino 2	Murcia		
Real Temprano 1	Monzón, Huesca; Ebro Valley		
Real Temprano 2	Monzón, Huesca; Ebro Valley		
San Ambrosio	Segorbe, Castellón; Valencian Community		
Santones	Calatayud, Zaragoza; Ebro Valley		
Tapalahoja	Lanjar, Almería; Andalucia		
Temprano Colomer	Logroño; Ebro Valley		
Temprano Gordo	Lebrija, Sevilla; Andalucia		
Toledo	Monzón, Huesca; Ebro Valley		
Velázquez	Abarán; Murcia		

[23], [49], [50]

### DNA extraction

#### Old material

After trying several DNA extraction commercial kits (Accuprep® GMO, Bioneer; Kit G2N10 Genelute™ Plant Genomic, Sigma; Realpure, Real; Invisorb® Spin Plant, Invitek) and modified DNA extraction methods available in the literature [10], [24]–[26] with both the testa and the endocarp tissues, we decided to use the protocol of Godoy and Jordano [26] with some modifications since this was the method that yielded the best results (data not shown). The maternal tissues forming the testa were separated from the rest of the seed (embryo) and treated with liquid nitrogen. Genomic DNA was extracted from 60 to 100 mg of testa and endocarp. Tissues were homogenized in 400 µl of extraction buffer (200 mM Tris-HCl pH 8.0; 70 mM EDTA; 2 mM NaCl; 20 mM sodium bisulfite) with a TissueLyser homogenizer (30 sec; 30 Hz). After homogenization, 85 µl of sarkosyl was added and the sample was incubated at 65°C for 30 min and centrifuged at 10,000 g for 20 min to remove insoluble material. In some cases this step had to be repeated for 5 additional min. DNA was precipitated by the addition of 95 µl of 10 M ammonium acetate and 200 µl of cold isopropanol; the mixture was centrifuged for 20 min at 16,000 g. The pellet was washed with 70% ethanol for 30 min, dried and resuspended in 100 µl MTE (modified TE) buffer (1M Tris-HCl pH 8.0; 0.5 M EDTA).

#### Young leaves

Genomic DNA extractions were performed as previously described [10] with some modifications. Approximately 50 mg of young leaves were treated with liquid nitrogen and homogenized with 300 µl of extraction buffer (100 mM Tris-HCl; 20 mM EDTA; 1.4 M NaCl; 2% CTAB, 1% PVP, 0.2% β-mercaptoethanol). The samples were incubated at 65°C for 30 min, mix with an equal volume of chloroform-isoamyl alcohol (24∶1) and centrifuged at 6,000 g for 15 min. The upper aqueous phase was recovered and mixed with 200 µl of cold isopropanol. The nucleic acid precipitation was recovered through centrifugation at 13,000 g for 5 min and washed in 400 µl of 10 M ammonium acetate. The pellet was washed with 70% ethanol for 30 min, dried and resuspended in 100 µl MTE buffer.

### DNA amplification

Extracted apricot genomic DNA was PCR-amplified using two sets of SSR loci developed in peach and apricot. The SSR set previously developed in peach and proved to be transferable to apricot [10] included 1 primer pair developed by Sosinski et al. [27] (pchgms3) and 5 by Cipriani et al. [28] (UDP96-001, UDP96-003, UDP96-008, UDP96-018 and UDP98-406). The other set was composed of 12 loci developed in apricot by Lopes et al. [29] (ssrPaCITA7, ssrPaCITA19, ssrPaCITA23, ssrPaCITA10, ssrPaCITA12, ssrPaCITA27) and by Messina et al. [30] (*UDAp-410*, *UDAp-411*, *UDAp-414*, *UDAp-415*, *UDAp-419*, *UDAp-420*) selected in base to the higher number of alleles per locus and heterozygosity.

Amplification reactions were carried out in 15 µL volumes containing 16 mM (NH_4_)_2_SO_4_, 67 mM Tris-HCl pH 8.8, 0.01% Tween20, 2 mM MgCl_2_, 0.1 mM each dNTP, 0.4 µM each primer, between 20–40 ng genomic DNA and 1 unit of BioTaq™ DNA polymerase (Bioline, London, UK) on an I-cycler (Bio-Rad Laboratories, Hercules, CA, USA) thermocycler using the following temperature profile: an initial step of 1 min at 94°C, 35 cycles of 30 s at 94°C, 30 s at 47/ 51/ 56/ 57°C (depending on each primer pair) and 1 min at 72°C, and a final step of 5 min at 72°C. Forward primers were labeled with a fluorescent dye on the 5′ end (Proligo, Paris, France). The PCR products were analyzed by capillary electrophoresis in a CEQ™ 8000 capillary DNA analysis system (Beckman Coulter, Fullerton, CA, USA). Samples were denaturalized at 90°C during 120 s, injected at 2.0 kV 30 s and separated at 6.0 kV during 35 min. Each reaction was repeated twice in each run to ensure size accuracy and to minimize run-to-run variation.

### Data analysis

For each SSR locus, allelic composition and the number of total alleles were determined in each accession. Putative alleles were indicated by the estimated size in bp. The program ARLEQUIN version 3.01 [31] was used to calculate the number of alleles per locus (A), observed heterozygosity (Ho), expected heterozygosity (He = 1-∑ *p_i_* ^2^ where p_i_ is the frequency of the i^th^ allele, [32]) and allele frequencies (considering *P*<0.05, p>0.9 as rare and fixed alleles respectively). POPGENE 1.32 software [33] was used to calculate the effective number of alleles (Ne = 1/1-He) and Wright's fixation index (F = 1-Ho/He) [34]. The probability of identity (PI = 1- ∑ *p_i_* ^4^ + ∑∑(2*p_i_p_j_*)^2^, where p_i_ and p_j_ are the frequency of the i^th^ and j^th^ alleles respectively) that measures the probability that two randomly drawn diploid genotypes will be identical assuming observed allele frequencies and random assortment [35] was calculated by IDENTITY 1.0 (Centre for Applied Genetics, University of Agricultural Sciences, Vienna, Austria).

The genetic relationships among the accessions studied were calculated using UPGMA cluster analysis of the similarity matrix obtained from the proportion of shared amplification fragments [36] with NTSYSpc 2.11 (Exeter Software, Stauket; NY, USA). The cophenetic correlation coefficient was computed for the dendrogram after the construction of a cophenetic matrix to measure the goodness of fit between the original similarity matrix and the dendrogram. Bootstrap support values were obtained from 2000 replicates using the program Treecon 1.3b [37].

Assignation of the genotypes to the different putative populations was studied with the program Structure 2.3.1 [38], [39], which identifies clusters of individuals on the basis of their genotypes at multiple loci using a Bayesian approach. Structure would attribute a probability Pr(X | *K*) given the data (X), and the logPr (X | *K*) is used to determine the more likely number of clusters [38]. The *k* value that provided the maximum likelihood over the runs was retained as the most probable number of clusters [40]. We used the admixture option and performed several runs of various lengths to infer the number of genetic clusters (*k*) represented by the individuals genotyped, testing all values of *k* from 1 to 10. Clustering solutions of the highest likelihood were obtained when most genomic assignments were distributed over 5 and 6 clusters. To choose the best value of k, for *k* = 5 and *k* = 6, ten independent replicates were run for 200,000 steps, after a burn-in period of 20,000 steps.

## Results

### DNA extraction and PCR amplification

In order to choose the best maternal tissue to obtain appropriate DNA for PCR amplification from the old stone collection, DNA was extracted and amplified from both testa and endocarp tissues. The results obtained showed a higher quality and repeatability of the amplifications with testa tissue (data not shown). Consequently, all the experiments were performed using the testa tissue. In the case of fresh apricot material from the *ex situ* collection, DNA was successfully recovered from leaves.

Repeatable amplifications were produced with DNA obtained from testa tissue of the old apricot cultivars with 13 of the 18 microsatellites assayed, four from peach (Pcghms3, UDP96-001, UDP96-008, UDP96-018) and 9 (ssrPaCITA7, ssrPaCITA19, ssrPaCITA23, ssrPaCITA10, ssrPaCITA12, ssrPaCITA27, *UDAp-414*, *UDAp-415*, *UDAp-420*) from apricot. Eleven of them (2 from peach and 9 from apricot) produced polymorphic repeatable amplifications with the 34 accessions from the stone collection and the 24 accessions from the ex situ collection (**Table 2**).

**Table 2:** List of the microsatellites that produced polymorphic repeatable amplification patterns among the genotypes studied.

Locus name	Reference	SSR motive	Predicted length (bp)	Size range (bp)	Annealing Temp (°C)
pchgms3	Sosinski et al. [27]	(CT)_19_	179	187–199	57
UDP96-001	Cipriani et al. [28]	(CA)_17_	120	110–112	57
ssrPaCITA7	Lopes et al. [29]	(AG)_22_	211	180–211	51
ssrPaCITA10	Lopes et al. [29]	(CT)_26_	175	158–179	47
ssrPaCITA12	Lopes et al. [29]	(TC)_16_	151	154–162	47
ssrPaCITA19	Lopes et al. [29]	(TC)_16_	114	112–156	51
ssrPaCITA23	Lopes et al. [29]	(AC)_2_(AG)_18_	146	141–156	51
ssrPaCITA27	Lopes et al. [29]	(TC)_8_ (TA)_6_(TG)_17_	262	227–256	47
*UDAp-414*	Messina et al. [30]	(AG)_21_	174	152–172	56
*UDAp-415*	Messina et al. [30]	(GA)_21_	156	150–160	56
*UDAp-420*	Messina et al. [30]	(CT)_20_	175	159–180	56

### Microsatellite diversity in the old stone collection

The 11 selected SSR loci produced polymorphic amplification fragments among the 34 analyzed apricot genotypes using DNA from testa tissues. The parameters of variability analyzed for these SSRs are presented in **Table 3**. A total of 47 alleles were detected, ranging from 2 (UDP96-001) to 7 (ssrPaCITA23), with an average of 4.27 alleles per locus. Allele frequencies ranged from 0.014 to 0.986 (mean = 0.244). Eight (17%) rare alleles were observed (*P*<0.05) but none was fixed (p≥0.9) in this collection. Some alleles were exclusive to certain genotypes. Thus ‘Acmé’ presented a unique allele at the ssrPaCITA10 locus, ‘Canino 2’ presented a unique allele at the pchgms3 locus, ‘De Hellin’ presented a unique allele at the ssrPaCITA10 locus, ‘Tapalahoja’ presented a unique allele at the ssrPaCITA27 locus and ‘Temprano Colomer’ presented a unique allele at the ssrPaCITA19 and *UDAp414* loci. All the selected microsatellites amplified one or two fragments per genotype and, consequently, they were considered as single locus SSRs.

**Table 3:** Genetic diversity parameters of the old and *ex situ* conserved genotypes analyzed in this study.

		OLD GENOTYPES						*EX SITU* CONSERVED GENOTYPES					
SSR	Size (bp)	A	Ne	PI	Ho	He	F	A	Ne	PI	Ho	He	F
ssrPaCITA7	187–223	5	2.69	0.28	0.36	0.64	0.42	2	1.38	0.64	0.33	0.32	−0.20
ssrPaCITA19	100–150	3	2.38	0.56	0.24	0.55	0.58	3	2.13	0.53	0.67	0.56	−0.41
ssrPaCITA23	136–156	7	5.43	0.11	0.65	0.84	0.21	5	3.11	0.25	0.63	0.73	0.08
ssrPaCITA10	147–179	5	2.64	0.34	0.34	0.63	0.45	3	2.86	0.34	0.71	0.67	−0.03
ssrPaCITA12	141–157	4	3.25	0.25	0.26	0.72	0.63	3	2.19	0.41	0.54	0.51	−0.07
ssrPaCITA27	246–264	5	2.29	0.34	0.31	0.59	0.39	3	1.83	0.51	0.50	0.43	−0.20
*UDAp-414*	150–214	4	2.90	0.30	0.42	0.67	0.35	3	1.67	0.54	0.33	0.35	0.07
*UDAp-415*	139–143	3	2.96	0.34	0.32	0.67	0.51	3	2.58	0.44	0.75	0.60	−0.29
*UDAp-420*	154–262	5	3.27	0.20	0.54	0.72	0.22	4	2.91	0.31	0.83	0.67	−0.33
pchgms3	220–240	4	2.52	0.40	0.56	0.61	0.07	3	1.92	0.48	0.54	0.50	−0.05
UDP96-001	108–128	2	1.78	0.60	0.29	0.44	0.33	2	1.80	0.61	0.38	0.49	0.25
**Mean**		4.27	2.91	0.39	0.39	0.64	0.38	3.09	2.22	0.46	0.56	0.54	−0.11

Observed heterozygosity ranged from 0.24 in ssrPaCITA10 and ssrPaCITA12 to 0.65 in ssrPaCITA23 (mean of 0.39). Expected heterozygosity ranged from 0.44 in UDP96-001 to 0.84 in ssrPaCITA23 (mean of 0.64). The comparison between the two parameters was carried out using the Wright's fixation index (F). For all the 11 loci analyzed this parameter was positive, meaning a deficit of heterozygotes. These results indicate a certain degree of inbreeding which could be explained by the fact that some genotypes could be genetically related. The maximum probability of identity was detected in UDP96-001 (0.60), with two alleles, and the minimum (0.11) in ssrPaCITA23, with seven alleles. The average was 0.39 and the total probability identity was 2.34×10^−6^. The value of Ne ranged from 1.78 (UDP96-001) to 5.43 (ssrPaCITA23) with an average of 2.91.

### Microsatellite diversity in the *ex situ* living collection

A set of 24 local Spanish apricot accessions conserved *ex situ* were analyzed with the same 11 loci described above in order to compare the diversity parameters with the material collected 60 years ago. The parameters of variability analyzed for these SSRs are presented in **Table 3**. A total of 34 alleles were detected, ranging from 2 (ssrPaCITA7, UDP96-001) to 5 (ssrPaCITA23), with an average of 3.09 alleles per locus. Allele frequencies ranged from 0.008 to 0.654 (mean = 0.29). Five (15%) rare alleles were observed (*P*<0.05) but none was fixed (p≥0.9). Some alleles were exclusive to certain genotypes. Thus ‘Ginesta’ presented a unique allele at the ssrPaCITA23 locus and ‘Cristali’ presented a unique allele at the ssrPaCITA27 locus. All the selected microsatellites amplified one or two fragments per genotype and consequently, they were considered as single locus SSRs.

Observed heterozygosity ranged from 0.33 in ssrPaCITA10 and ssrPaCITA12 to 0.83 in *UDAp420* (mean of 0.56). Expected heterozygosity ranged from 0.32 in UDP96-001 to 0.73 in ssrPaCITA23 (mean of 0.54). The Wright's fixation index (F) was positive for 3 loci, whereas for the other 8 loci this parameter was negative, indicating a higher observed than expected heterozygosity. The maximum probability of identity was detected in UDP96-001 (0.60), with four alleles, and the minimum (0.11) in ssrPaCITA23, with 7 alleles. The average was 0.34 and the total probability identity was 2.42 x 10^−6^. The value of Ne ranged from 1.38 (ssrPaCITA7) to 3.11 (ssrPaCITA23) with an average of 2.22.

### Identification of the different accessions

The different amplification fragment combinations obtained with 11 SSRs allowed us to distinguish 34 unique genetic profiles among the genotypes of the old stone collection revealing five pairs of homonymous accessions: ‘Canino 1’ and ‘Canino 2’, ‘Real Temprano 1’ and ‘Real Temprano 2’, ‘Giletano 1’ and ‘Giletano 2’, ‘Real Fino 1’ and ‘Real Fino 2’ and ‘Blanco de Murcia 1’ and ‘Blanco de Murcia 2’. No synonymies were found in the material studied. The range of alleles sizes obtained in this work was similar to those reported for the same SSRs in peach [27]–[30], [41]. Regarding the living *ex situ* collection a total of 15 unique genetic profiles were revealed with 4 synonymies and 6 homonymies.

When the results obtained from the stone collection were compared with the *ex situ* apricot germplasm collections only one of the genotypes of the stone collection analyzed in this work (‘Canino 2’) had the same genotype profile than two other genotypes in the living *ex situ* collection (‘Canino 1’ and ‘Canino 2’) suggesting that the rest of the old genotypes are no longer conserved in the *ex situ* collections analyzed.

### Similarity relationships and clustering

The dendrogram generated from UPGMA cluster analysis based on the Nei and Li similarity index for the old apricot collection showed a cophenetic correlation coefficient of 0.69 that corresponds to a good fit between the cophenetic and the similarity matrixes. Due the lack of information on the actual geographic origin of some of the samples we decided to analyze the population structure of this material. Using the whole set of loci in the cluster analysis with the Structure software, the highest likelihood was observed for *k* = 5. The estimated membership of each individual to each cluster did not correspond to the site of collection for every group of samples. In some cases, a group was formed by multiple genotypes with genomes composed by some diverse fractions of clusters. In this case, additional subclustering runs of Structure were required. These runs used only those individuals that were assigned to that cluster previously, with 20,000 interactions with a burn-in period of 5,000. For subclustering runs, k equaled the number of the genotypes associated with the cluster. Although clustering solutions differed across runs, the same individuals tended to be misclassified across runs.

**Figure 1:**
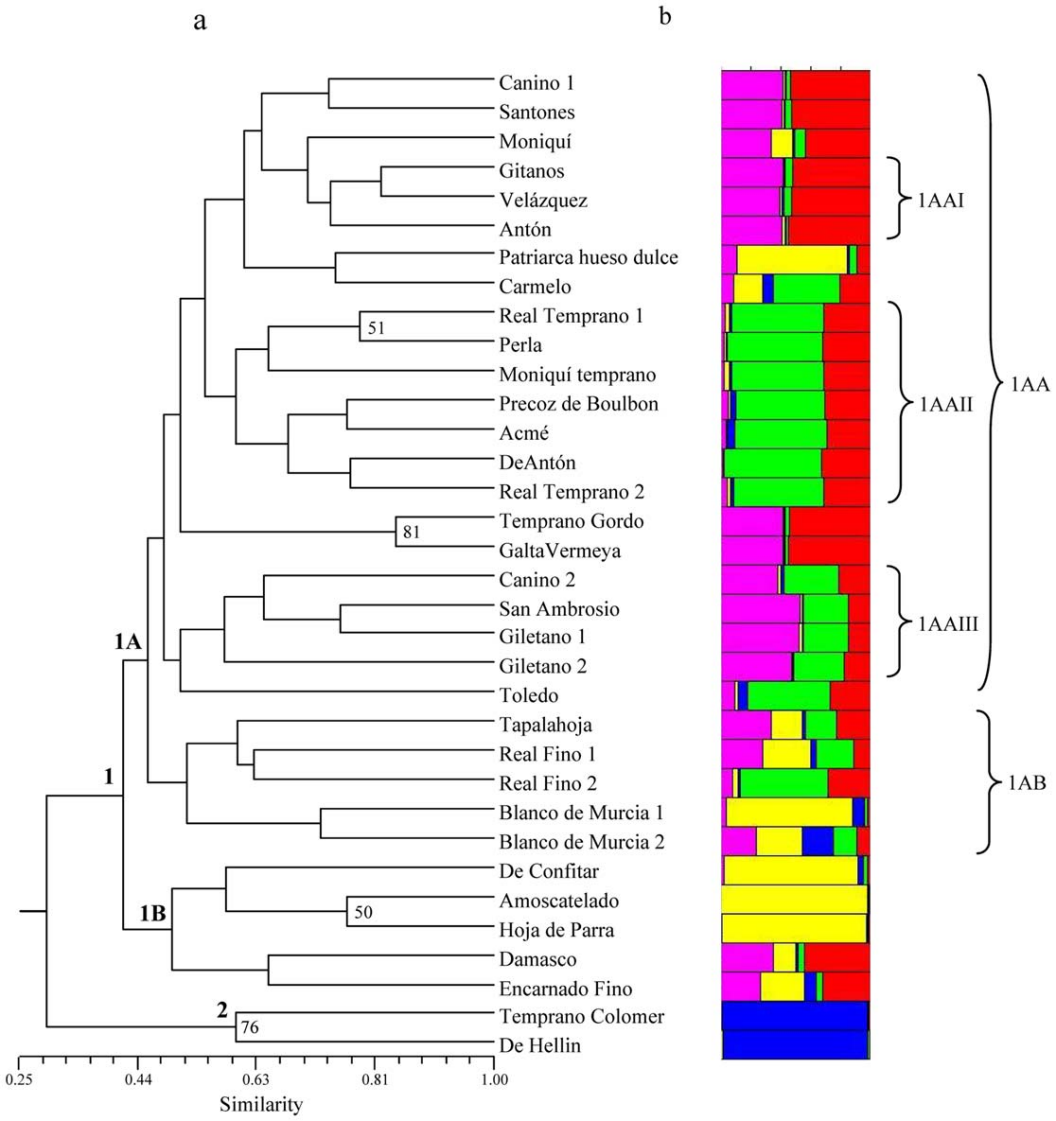
Clustering of 34 apricot accessions from an old stone collection. (A) Dendrogram based on UPGMA analysis using the similarity matrix generated by the Nei and Li coefficient after amplification with 11 pairs of microsatellite primers. (B) Representative estimate of population structure. The plot represent the highest-likelihood run among 10 Structure runs with *k* = 5 putative populations, represented by different colours.

The UPGMA dendrogram (**Figure 1**) shows one main group and two accessions (‘De Hellin’ and ‘Temprano Colomer’), that clearly separate from the rest of the genotypes. Results from the Structure software clearly show how these two genotypes belong to a different and defined subgroup separated from the rest of the genotypes. Regarding the main group, in general, a mix of genotypes collected from different locations can be observed. In this main cluster two groups (1A and 1B) can be defined. In group 1B five genotypes collected from the Ebro Valley region (Huesca, Logroño and Zaragoza) are clustered together and this is supported by the results from the Structure software, in which we can see how three of these genotypes show a similar fraction of their genomes belonging to the same cluster. In 1A, there are two subgroups: 1AA and 1AB. In the first subgroup (1AA) a mix from different collection sites are clustered, although some clear subgroups collected in the same region [1AAI (Murcia), 1AAII (Ebro Valley) and 1AAIII (Valencian Community)] which present similar population structure can be differentiated. In subgroup 1AB, 75% of the clustered genotypes were collected in the same region (Ebro Valley).

### Combined analysis

**Figure 2:**
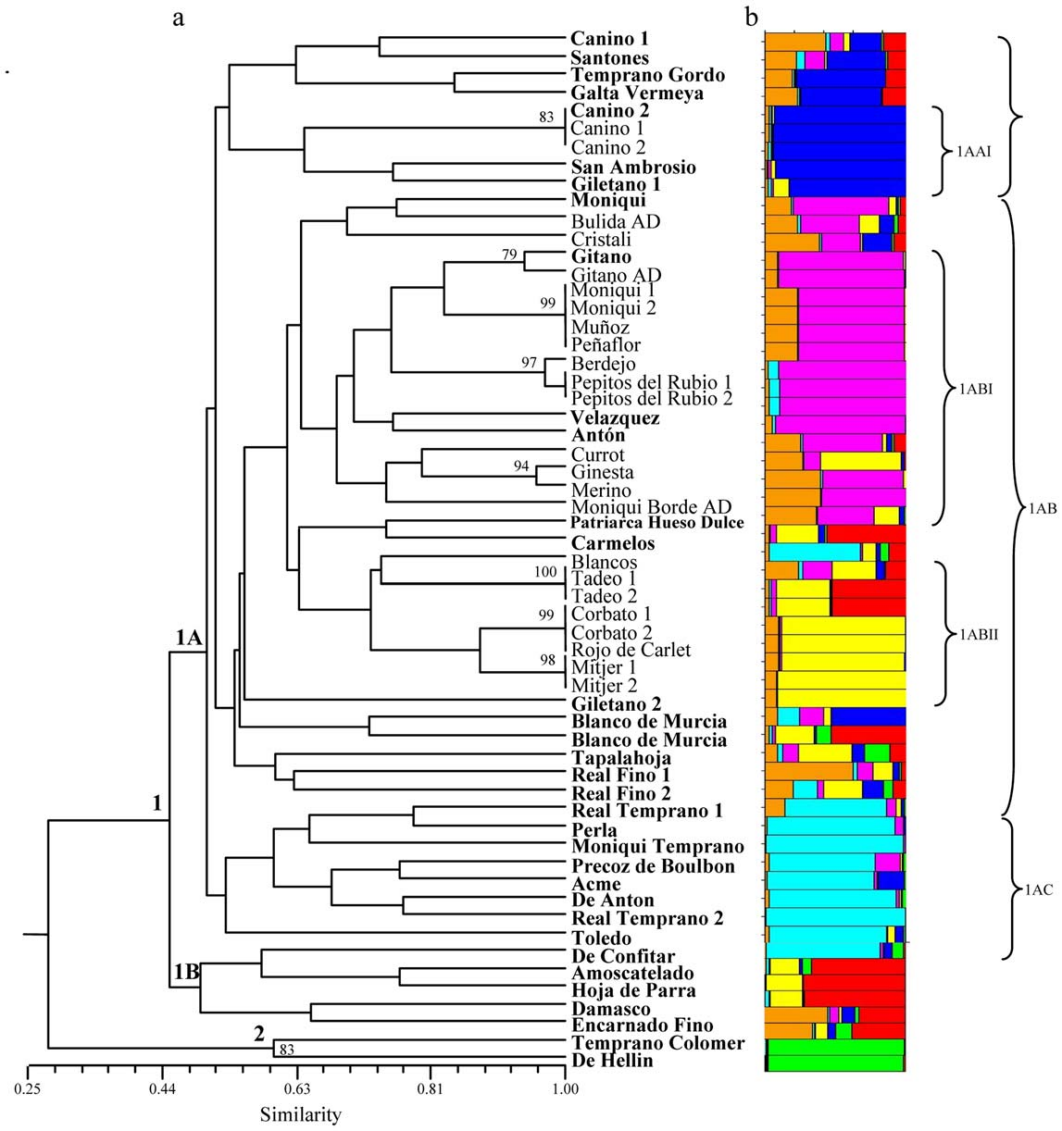
Clustering of 58 apricot accessions from both, old stone and *ex situ* collections. (A) Dendrogram based on UPGMA analysis using the similarity matrix generated by the Nei and Li coefficient after amplification with 11 polymorphic SSR loci. The accesions from the stone collection are represented in bold; the rest are the accessions conserved *ex situ*. (B) Representative estimate of population structure. The plot represent the highest-likelihood run among ten Structure runs with *k* = 7 putative populations, represented by different colours.

When both the *ex situ* collection and the old stone collection are analyzed together, again the two accessions from the old material that are clearly different from the rest (‘De Hellin’ and ‘Temprano Colomer’), are separated from the main group of genotypes (see **Figure 2**). Excluding these two accessions, the main cluster (cluster 1) can be divided in two groups: 1A and 1B. All the genotypes in cluster 1B belong to the old stone collection. In cluster 1A three groups can be distinguished (1AA, 1AB and 1AC). All the genotypes in subgroup 1AC belong to the old stone collection and a mixture of genotypes from both collections are clustered in 1AA and 1AB although in both groups the genotypes from each collection tend to cluster together.

## Discussion

The results obtained in this work show that DNA of sufficient quality for PCR analysis and fingerprinting purposes can be obtained from old apricot seeds stored during 60 years at room temperature.

### DNA extraction from old stones

Since DNA degradation is very common in old samples [42]–[44], the first objective of this work was to optimize the DNA extraction method of maternal tissue present in old apricot stones in order to get successful results. After trying different methods the best results with both DNA from endocarp and testa tissues was obtained following the protocol described in [26] with some modifications. Although amplification was obtained with both tissues, the amplifications with testa tissue showed a higher quality and repeatability and, consequently, all the experiments were performed using that tissue. The lower DNA quality and repeatability of the amplifications from the endocarp tissue could be due to DNA degradation that can be faster in the endocarp that in the testa since the endocarp is more exposed to external degrading agents. Moreover, the endocarp is a woody tissue with lower DNA quantity than other plant tissues.

### SSR polymorphism and genetic diversity

The results obtained in this work show that microsatellites can be effectively used for fingerprinting purposes using old apricot plant material. Amplification was successful with the 11 selected SSR loci developed in apricot and peach, distinguishing 34 unique genetic profiles in the old collection. The use of the approach described in this work is supported by the fact that some of the cultivars with the same name from the old stone and the *ex situ* collection, as ‘Canino 1’ and ‘Canino 2’ from the *ex situ* collection and ‘Canino 2’ from the stone collection, were identical in their allelic composition.

The mean value of 4.27 alleles per locus obtained was higher than the 3.10 alleles per locus reported previously with 11 SSRs and 40 cultivars from different areas around the world [11]. It was similar to the 4.1 alleles per locus reported with 20 SSRs and 48 genotypes from diverse geographical areas [10] and the 4 alleles per locus obtained with 36 accessions from different areas of Murcia (Spain) [17]. However, it was lower than the value of 7.64 alleles per locus in 74 cultivars analyzed with 12 loci [12], 12.3 alleles per locus in 44 cultivars [16] and 13.3 alleles per locus in 133 accessions [13]. These results are expected taking into account that the accessions analyzed in our work were just of Spanish origin, but highlight a wider variability with fewer samples than in currently preserved *ex situ* living collections.

### Clustering and population structure

A reduction in the number of alleles was observed in the *ex situ* collections when compared to the old material. This could reveal a loss of alleles over time because some of the traditional varieties have disappeared. Varieties are unique combinations of alleles. It is possible that some, most, or all the alleles of an extinct variety can be present in a different conserved variety, although not in that particular combination [45], resulting in the irreversible loss of selected appropriate allele combinations. In apricot, currently about 10 main varieties are cultivated and commercialized in Spain [46] although more than 100 accessions, most of them foreign cultivars, are conserved in different *ex situ* collections [47], [48]. However, only one (‘Canino 2’) of the genotypes of the old collection is currently conserved in the *ex situ* collections analyzed.

UPGMA analysis of our set of apricot accessions from the old collection produced groups that were not generally based on the site of collection, in the cases in which this information was available, and, similarly, the old collection analyzed does not have a clear population structure. This could probably be due to the exchange of plant material among the different apricot growing regions and to the fact that the collection analyzed is only a fraction of all apricot cultivars in Spain 60 years ago. This is also the case when both the *ex situ* collection and the old stone collection are analyzed together. However, some exceptions were found; in some cases accessions collected in the same region clustered together; examples include the groups 1AAI (collected in Murcia), 1AAIII (collected in Valencian Community) and 1AAII, 1AB, 1B with more than 80% of genotypes from the Ebro Valley region (see **Figure 1**). This could reveal a common origin of those groups probably by seed propagation before grafting was a widespread technique in the apricot growing areas. These results from the UPGMA analysis were supported by the Bayesian clustering method.

In the comparison of both collections most of the accessions from the old collection cluster together in different subgroups (see **Figure 2**). Almost all of these, are lost cultivars not similar to other cultivars from the *ex situ* collection analysed in this work, at least with the SSR loci used, and alert on the cultivars that should be prioritized for prospection and conservation. Although additional germplasm collections should be studied to check for the presence of these old varieties, this work can be considered as a window to the past and an effort should be made to try to recover those cultivars that could still be present in small villages or in familiar orchards in rural areas since the information where the cultivars were collected is still available for most cases.

This case study in apricot shows that the approach used in this work can also be most useful to study the loss of genetic diversity and the genetic erosion that has taken place in other species and in other areas of the world, where old seeds or endocarps are still available. This is plausible, since stone collections have been used for morphological identification purposes and are much easier to preserve that living trees. Moreover, the results obtained in this work show that genetic profiles can be obtained from that kind of material kept without particular preservation requirements.

## References

[1] Van de Wouw M, Kik C, van Hintum T, van Treuren R, Visser B (2009). Genetic erosion in crops: concept, research results and challenges.. Plant Genetic Resour.

[2] Tanksley SD, McCouch SR (1997). Seed banks and molecular maps: Unlocking genetic potential from the wild.. Science.

[3] Esquinas-Alcazar J (2005). Protecting crop genetic diversity for food security: political, ethical and technical challenges.. Nat Rev Genet.

[4] Jarvis I, Brown A, Hung C, Collado L, Latournerie L (2008). A global perspective of the richness and evenness of traditional crop-variety diversity maintained by farming communities.. P Natl Acad Sci U S A.

[5] Khoury C, Laliberté B, Guarino L (2010). Trends in *ex situ* conservation of plant genetic resources: a review of global crop and regional conservation strategies.. Genet Resour Crop Ev.

[6] Arumuganathan K, Earle E (1991). Nuclear DNA content of some important plant species.. Plant Mol Biol Rep.

[7] Hormaza JI, Yamane H, Rodrigo J, Kole C (2007). Apricot.. Genome Mapping and Molecular Breeding in Plants.

[8] Layne REC, Bailey CH, Hough LF, Janick J, Moore JN (1996). Apricots.. Fruit Breeding, vol II: Tree and Tropical Fruits.

[9] Faust M, Suranyi D, Nyujto F (1998). Origin and dissemination of apricot.. Hort Rev.

[10] Hormaza JI (2002). Molecular characterization and similarity relationships among apricot (*Prunus armeniaca* L.) genotypes using simple sequence repeats.. Theor Appl Genet.

[11] Romero C, Pedryc A, Muñoz V, Llacer G, Badenes ML (2003). Genetic diversity of different apricot geographical groups determined by SSR markers.. Genome.

[12] Zhebentyayeva TN, Reighard GL, Gorina VM, Abbott AG (2003). Simple sequence repeat (SSR) analysis for assessment of genetic variability in apricot germplasm.. Theor Appl Genet.

[13] Maghuly F, Fernandez EB, Ruthner SZ, Pedryc A, Laimer M (2005). Microsatellite variability in apricots (*Prunus armeniaca* L.) reflects their geographic origin and breeding history.. Tree Genet Genomes.

[14] Sánchez-Pérez R, Ruiz D, Dicenta F, Egea J, Martínez-Gómez P (2005). Application of simple sequence repeat (SSR) markers in apricot breeding: molecular characterisation, protection and genetic relationships.. Sci Hortic.

[15] Sánchez-Pérez R, Martínez-Gómez P, Dicenta F, Egea J, Ruiz D (2006). Level and transmission of genetic heterozygosity in apricot (*Prunus armeniaca* L.) explored using simple sequence repeat markers.. Genet Resour Crop Ev.

[16] Khan M, Maghuly F, Borotto-Fernandez EG, Pedryc A, Katinger H (2008). Genetic diversity and population structure of apricot (*Prunus armeniaca* L.) from Northern Pakistan using Simple Sequence Repeats.. Silvae Genet.

[17] Martínez-Mora C, Rodríguez J, Cenis JL, Ruiz-García L (2009). Genetic variability among local apricots (*Prunus armeniaca* L.) from the Southeast of Spain.. Span J Agric Res.

[18] Pedryc A, Ruthner S, Herman R, Krska B, Hegedüs A (2009). Genetic diversity of apricot revealed by a set of SSR markers from linkage group G1.. Sci Hortic.

[19] Krichen L, Bourguiba H, Audergon JM, Trifi-Farah N (2010). Comparative analysis of genetic diversity in Tunisian apricot germplasm using AFLP and SSR markers.. Sci Hortic.

[20] Zohary D, Hopf M (1993). Domestication of plants in the Old World (2nd edition)..

[21] Hagen LS, Khadari B, Lambert P, Audergon JM (2002). Genetic diversity in apricot revealed by AFLP markers: species and cultivar comparisons.. Theor Appl Genet.

[22] De Vicente MC, Truco MJ, Egea J, Burgos L, Arús P (1998). RFLP variability in apricot [*Prunus armeniaca* (L.)].. Plant Breeding.

[23] Herrero J (1964). Cartografia de Frutales de Hueso y Pepita..

[24] Fulton TM, Chunwongse J, Tanksley SD (1995). Microprep protocol for extraction of DNA from tomato and other herbaceous plants.. Plant Mol Biol Rep.

[25] Cheng FS, Brown SK, Weeden NF (1997). A DNA extraction protocol from various tissues in woody species.. Hortscience.

[26] Godoy JA, Jordano P (2001). Seed dispersal by animals: exact identification of source trees with endocarp DNA microsatellites.. Mol Ecol Notes.

[27] Sosinski B, Gannavarapu M, Hager LD, Beck LE, King GJ (2000). Characterization of microsatellite markers in peach [*Prunus persica* (L.) Batsch].. Theor Appl Genet.

[28] Cipriani G, Lot G, Huang W-G, Marrazzo MT, Peterlunger E (1999). AC/GT and AG/CT microsatellite repeats in peach [*Prunus persica* (L) Batsch]: isolation, characterisation and cross-species amplification in *Prunus.*. Theor Appl Genet.

[29] Lopes MS, Sefc KM, Laimer M, Da Câmara Machado A (2002). Identification of microsatellite loci in apricot.. Mol Ecol Notes.

[30] Messina R, Lain O, Marrazzo MT, Cipriani G, Testolin R (2004). New set of microsatellite loci isolated in apricot.. Mol Ecol Notes.

[31] Excoffier L, Laval G, Schneider S (2005). Arlequin ver. 3.0: An integrated software package for population genetics data analysis.. Evolutionary Bioinformatics Online.

[32] Nei M (1973). Analysis of gene diversity in subdivided populations.. P Natl Acad Sci U S A.

[33] Yeh FC, Young RC, Timothy B, Boyle TBJ, Ye ZH (1997). Popgene, the user-friendly shareware for population genetic analysis..

[34] Wright S (1951). The genetical structure of populations.. Ann Eugenics.

[35] Paetkau D, Calvert W, Stirling I, Strobeck C (1995). Microsatellite analysis of population structure in Canadian polar bears.. Mol Ecol.

[36] Nei M, Li WH (1979). Mathematical-model for studying genetic-variation in terms of restriction endonucleases.. P Natl Acad Sci U S A.

[37] Van de Peer Y, De Watchter R (1994). TREECON for Windows: A software package for the construction and drawing of evolutionary trees for the Microsoft Windows environment.. Computer Applications in the Bioscience.

[38] Pritchard JK, Stephens M, Donnelly P (2000). Inference of population structure using multilocus genotype data.. Genetics.

[39] Falush D, Stephens M, Pritchard JK (2003). Inference of population structure using multilocus genotype data: linked loci and correlated allele frequencies.. Genetics.

[40] Pritchard JK, Wen W (2004). Documentation for the STRUCTURE software Version 2.. http://www.pritch.bsd.uchicago.edu/software/structure2_1.html.

[41] Testolin R, Marrazo T, Cipriani G, Quarta R, Verde I (2000). Microsatellite DNA in peach [*Prunus persica* (L.) Batsch] and its use in fingerprinting and testing the genetic origin of cultivars.. Genome.

[42] Gugerli F, Parducci L, Petit RJ (2005). Ancient plant DNA: review and prospects.. New Phytol.

[43] Walters C, Reilley AA, Reeves PA, Baszczak J, Richards CM (2006). The utility of aged seeds in DNA banks.. Seed Sci Res.

[44] Leino MW, Hagenblad J, Edqvist J, Strese EMK (2009). DNA preservation and utility of a historic seed collection.. Seed Sci Res.

[45] Fowler C, Mooney P (1990). Shattering: Food, politics, and the loss of genetic diversity..

[46] Rodrigo J, Hormaza JI (2005). El albaricoquero.. Diversidad genética y situación general del cultivo.

[47] Egea-Sánchez JM, Avilés I, Egea-Fernández JM (2008). Inventario y catalogación de variedades locales de la Región de Murcia..

[48] Badenes ML, Martínez-Calvo J, García-Carbonel S, Villarubia D, Llácer G (1997). Descripción de variedades autóctonas valencianas de albaricoquero..

[49] Badenes ML, Asíns MJ, Carbonell EA, Llácer G (1996). Genetic diversity in apricot, *Prunus armeniaca*, aimed at improving resistance to plum pox virus.. Plant Breeding.

[50] Got N (1958). L'abricotier..

